# Novel Morphological Classification of Intracranial Aneurysm Wall Irregularity Associates Specific Features with Increased Size and Rupture Risk: A Retrospective Single-Center Cross-Sectional Study

**DOI:** 10.3390/neurolint18070126

**Published:** 2026-06-29

**Authors:** Kamil Krystkiewicz, Aleksander Kowal, Magdalena Krystkiewicz-Orzechowska, Filip Arczewski, Karol Dziedzic, Marcin Tosik

**Affiliations:** 1 Department of Neurosurgery and Neurooncology, Copernicus Memorial Hospital, 93-513 Lodz, Poland; aleksanderwkowal@gmail.com (A.K.); farczewski@interia.pl (F.A.); karol.dziedzic@student.umed.lodz.pl (K.D.); marcin.tosik@wp.pl (M.T.); 2 Department of Molecular Carcinogenesis, Medical University of Lodz, 92-215 Lodz, Poland; magdalena.orzechowska@umed.lodz.pl

**Keywords:** intracranial aneurysm, wall irregularity, aneurysm morphology, rupture risk, PHASES, ELAPSS, UIATS, subarachnoid hemorrhage

## Abstract

Introduction/Objectives: Wall irregularity is a known risk factor in the evaluation of intracranial aneurysms, but the prognostic value of its subtypes remains unclear. Materials and methods: In this retrospective single-center cross-sectional study (2023–2025), we reviewed consecutive adult patients with intracranial aneurysms. Morphology was classified as daughter sac, multilobulated, or complex irregularity. We compared rupture status and calculated PHASES, ELAPSS, and UIATS scores. Principal Component Analysis (PCA), and logistic and linear regression were applied. Results: A total of 180 patients with 180 index aneurysms were included; mean age was 67.2 ± 12.1 years, and 72.2% were women. Overall, 43.3% of aneurysms were irregular, specifically: daughter sac (25.0%), multilobulated (36.1%), and complex irregularity (11.1%). SAH occurred in 40 patients (22.2%). Ruptured aneurysms had larger maximum diameter, size ratio, and aspect ratio (all *p* < 0.0001), plus higher 5-year PHASES (*p* = 0.0091) and ELAPSS growth scores (*p* < 0.0001). PCA identified three clusters with differing 5-year rupture risks; Cluster 3 had the highest risk (5.71 ± 5.25%) and was characterized by a higher proportion of daughter sac and multilobulated morphology (*p* = 1.65 × 10^−7^ and 8.80 × 10^−16^). Linear models showed each irregular subtype was associated with significantly larger aneurysm size. Conclusions: Irregular wall patterns were common and associated with larger aneurysm dimensions and higher risk scores. These findings support further investigation of refined morphological descriptors in rupture risk stratification.

## 1. Introduction

The management of intracranial aneurysms involves a complex, multifactorial decision-making process aimed at preventing subarachnoid hemorrhage (SAH), a potentially devastating complication. Several risk stratification models, including PHASES, ELAPSS, and UIATS, support therapeutic decisions; however, significant uncertainty remains, and final recommendations often depend on clinical judgment [1,2,3,4,5]. Treatment options include microsurgical clipping and endovascular coiling, both well-established and complementary strategies in modern neurovascular care. Despite their efficacy, these interventions carry a risk of serious complications [5,6,7]. Aneurysm wall irregularity is one of the morphological features frequently considered in treatment decisions. In the Unruptured Cerebral Aneurysm Study of Japan (UCAS Japan), the presence of an irregular aneurysmal sac was associated with a higher risk of rupture [8]. Similar findings were reported by Lindgren et al. in a Finnish cohort [9]. Nonetheless, neither PHASES nor ELAPSS incorporates aneurysm morphology as a predictive factor, while the UIATS model includes both irregularity and lobulated shape in its morphological criteria. However, both the UCAS [8] and Lindgren [9] studies were conducted in populations (Japanese and Finnish, respectively) known to have a higher baseline risk of rupture, limiting the generalizability of their findings [2,3]. Despite the recognized clinical relevance of aneurysm wall irregularity, several important gaps remain. First, most previous studies have evaluated irregularity as a binary feature, classified simply as present or absent, without accounting for the morphological heterogeneity of irregular aneurysm walls. Second, terms such as daughter sac, bleb, lobulation, and multilobulated morphology are inconsistently used across studies, limiting reproducibility and comparison between cohorts. Third, it remains unclear whether specific irregularity patterns are associated with aneurysm size, established risk scores, or rupture status to the same extent.

To address this gap, we evaluated aneurysm wall irregularity using both an overall regular-versus-irregular classification and a feature-based assessment of specific morphological descriptors, including daughter sac, multilobulated morphology, and complex irregularity. We hypothesized that specific irregularity patterns would be associated with larger aneurysm dimensions and higher estimates of rupture or growth risk. Therefore, this study aimed to assess the prevalence of these morphological descriptors and examine their relationship with clinical characteristics; aneurysm morphometry; rupture status; and established risk assessment tools, including PHASES, ELAPSS, and UIATS.

## 2. Materials and Methods

### 2.1. Patient Cohort

We conducted a retrospective, single-center, cross-sectional analysis of consecutive adult patients diagnosed with and treated for ruptured or unruptured intracranial aneurysms in the Department of Neurosurgery at M. Copernicus Memorial Hospital in Lodz, Poland, from January 2023 to January 2025. Medical records and radiological documentation were reviewed to obtain the clinical and imaging variables required for PHASES, ELAPSS, and UIATS scoring. UIATS was calculated retrospectively using all available clinical records and, when present, prior imaging. For UIATS components requiring longitudinal information, including documented aneurysm growth on serial imaging and de novo aneurysm formation, points were assigned only when these findings were explicitly documented in the medical record or on prior imaging. In the absence of prior imaging or documented evidence of growth or de novo aneurysm formation, these items were treated as not contributing to the UIATS score; no imputation was performed. During the study period, 198 consecutive adult patients with intracranial aneurysms were screened for eligibility. Information on hereditary disorders associated with intracranial aneurysm formation, including autosomal dominant polycystic kidney disease, Marfan syndrome, and other documented connective-tissue or familial aneurysm syndromes, was extracted from the medical records. Patients with documented hereditary aneurysm-associated disorders were excluded from the final analysis to reduce confounding related to inherited aneurysm predisposition. Consequently, the autosomal dominant polycystic kidney disease component of UIATS did not contribute to the scores in the included cohort. A total of 18 patients were excluded: 13 for incomplete or non-diagnostic imaging data that precluded reliable assessment of aneurysm morphology; 3 for missing essential clinical or radiological variables required for risk-score calculation; and 2 for known hereditary disorders associated with intracranial aneurysm formation, including autosomal dominant polycystic kidney disease and Marfan syndrome. The final study cohort consisted of 180 patients.

The radiological assessment involved a detailed evaluation of aneurysm shape, size, and position, based on high-resolution CT angiography (CTA) or digital subtraction angiography (DSA). CTA was performed on a 64-detector CT scanner (SOMATOM Definition AS+, Siemens, Erlangen, Germany) from the C2 level to the vertex after injecting 70–90 mL of iodinated contrast (350 mg I/mL) at 4–5 mL/s, using bolus-tracking in the carotid siphon. Images were reconstructed with 0.5–0.75 mm slices using soft-tissue and vascular kernels, with multiplanar reconstructions (MPRs), maximum intensity projections (MIPs), and morphometric assessments via INFINITT PACS 7.0 (INFINITT Healthcare, Seoul, Republic of Korea). Diagnostic DSA employed a biplane angiographic system (Azurion 7 B20/20, Philips, Best, The Netherlands), with bilateral injections of 5–7 mL of nonionic contrast at 3–5 mL/s, at a frame rate of 3–4 fps. Standard anterior–posterior and lateral views were complemented by customized angles and magnifications. Three-dimensional rotational angiography (200° rotation, ~200 frames) was performed for morphometry, measuring neck width, dome height, and wall irregularities such as daughter sacs or lobulations. Morphological irregularities were classified as daughter sacs, multilobulated, or complex. The maximum diameter was measured on CTA or DSA in the plane that showed the widest cross-section of the aneurysm, from outer wall to outer wall. When daughter sacs or lobulations were present, the protruding segment was included in the measurement. To improve reproducibility, two independent researchers (KK and AK) performed the measurements. Morphological and morphometric assessments were performed separately and independently by two assessors using anonymized CTA and/or DSA datasets. The assessors were not provided with calculated PHASES, ELAPSS, or UIATS scores, or with treatment recommendations, at the time of image review. Disagreements in the four-category morphological classification were resolved by consensus review after completion of the independent assessments. Interobserver agreement was quantified using percentage agreement and Cohen’s kappa. Because the study was retrospective and included ruptured aneurysms, complete blinding to rupture status could not be guaranteed in all cases, particularly when imaging findings or the clinical imaging context suggested acute subarachnoid hemorrhage.

The study was reviewed by the Bioethics Committee at the Institute “Polish Mother’s Memorial Hospital” in Lodz, Poland, which issued Statement No. KB-41/2026 on 5 May 2026, confirming that the retrospective study did not constitute a medical experiment under Polish law and therefore did not require formal ethics committee approval. All patients provided written informed consent for the use of anonymized data for research purposes.

### 2.2. Statistical Analysis

Data distribution was assessed using the Shapiro–Wilk test. Continuous variables are presented as means with standard deviations (mean ± SD), and categorical variables are presented as frequencies and percentages. Between-group comparisons of continuous variables were performed using the Mann–Whitney U test. Associations between categorical variables were evaluated using the chi-square test or Fisher’s exact test, as appropriate, based on expected cell counts.

To assess the relationship between morphological features and continuous outcomes, multivariable linear regression models were used. Independent variables included predefined aneurysm morphological features (e.g., daughter sac, multilobulation, complex irregularity), and the dependent variable was the maximum aneurysm diameter. Regression coefficients, along with their corresponding 95% confidence intervals (CIs) and *p*-values, were reported.

Multivariable logistic regression models were constructed to evaluate the association between morphological features and SAH while adjusting for potential confounders, including aneurysm size.

To mitigate selection bias and confounding in observational data, propensity score matching (PSM) was used. Propensity scores were estimated using logistic regression, with age, sex, and PHASES score as covariates. Nearest-neighbor matching without replacement was performed in a 1:1 ratio using a caliper of 0.2 standard deviations of the logit of the propensity score. After matching, 40 pairs were retained (*n* = 80). Covariate balance before and after matching was assessed using standardized mean differences and visual inspection of covariate distributions. After matching, covariate balance between groups was assessed using standardized mean differences (SMDs) and visual inspection of covariate distributions. Post-matching comparisons between ruptured and unruptured aneurysms were performed using the Mann–Whitney U test for continuous variables and the chi-square test or Fisher’s exact test for categorical variables, as appropriate. Principal Component Analysis (PCA) was applied to explore latent patient subgroups based on a combination of morphometric and morphological variables. Before PCA, we employed a generalized additive model (GAM) with a thin-plate spline smoother to identify the most informative predictors of 5-year rupture risk (PHASES score). Variables with a GAM *p* < 0.05 were retained for subsequent analysis, thereby providing data-driven feature selection and reducing noise from collinear or weakly associated parameters. PCA is a dimensionality-reduction technique that transforms a set of possibly correlated quantitative variables into a smaller number of uncorrelated principal components, each capturing the maximum possible variance in the data. By projecting the original variables into this low-dimensional space, PCA enhances visualization, mitigates multicollinearity, and simplifies downstream statistical modeling. In our study, continuous morphometric measures (e.g., maximum diameter, size ratio) were standardized and entered the PCA; categorical morphology descriptors (daughter sac, multilobulated, complex irregularity) were first dummy-encoded so they could be analyzed alongside the quantitative features. The patient coordinates on the first two PCA dimensions were then subjected to agglomerative hierarchical clustering. The patient coordinates on the first two PCA dimensions were then subjected to agglomerative hierarchical clustering using Ward’s linkage and Euclidean distance. The selection of three clusters (k = 3) was based on an exploratory visual inspection of the PCA biplot. The distribution of data points along PC1 and PC2 suggested three apparent groupings. All analyses were conducted in the R environment (version 4.4.2) using packages such as FactoMineR, factoextra, ggplot2, tidyverse, gtsummary, and dplyr [10]. Two-sided *p*-values < 0.05 were considered statistically significant. All statistical analyses were performed using Stata version 18.5 (StataCorp, College Station, TX, USA) and the R environment (version 4.4.2). Missing data were handled using an available-case approach for descriptive analyses and a complete-case approach for regression, propensity score matching, and PCA-based analyses. No statistical imputation was performed. For categorical variables with missing observations, the number of missing values was reported, and percentages were calculated using the available denominator unless otherwise specified. Variables with substantial missingness, particularly smoking status, were not entered into multivariable regression models or propensity score matching. Patients with missing values in variables required for a specific model were excluded only from that analysis.

### 2.3. Classification of Aneurysm Morphology

Aneurysm sac morphology was assessed using both an overall binary classification and a feature-based morphological classification. First, each index aneurysm was classified as regular or irregular according to the overall regularity of the aneurysm wall contour. Aneurysms were classified as irregular if any deviation from a smooth, continuous wall contour was observed, including focal protrusions, lobulations, sac asymmetry, or a combination of these features.

**Figure 1:**
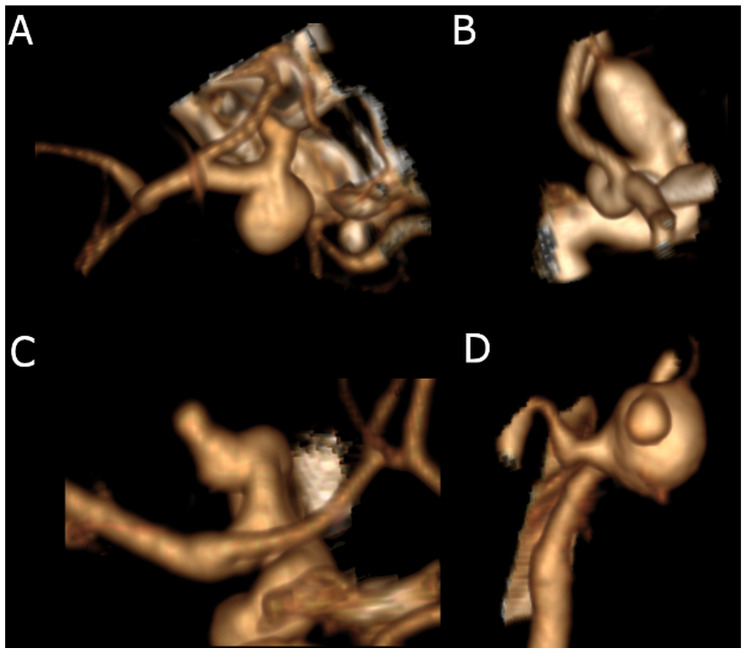
Morphological classification of intracranial aneurysm wall irregularity. (**A**) Regular shape aneurysm. (**B**) Daughter sac: a localized protrusion involving < 50% of the parent sac diameter. (**C**) Multilobulated: lobulations encompassing ≥ 50% of the sac contour, indicating broader and more complex geometry. (**D**) Complex irregularity: coexistence of a daughter sac arising from an already multilobulated aneurysm, representing the highest grade of wall irregularity.

Second, irregular aneurysms were categorized by dominant morphological pattern as daughter sac, multilobulated, or complex irregularity. A daughter sac was defined as a focal protrusion involving less than 50% of the primary sac dimension. Multilobulated morphology was defined as lobulations involving 50% or more of the sac contour without a distinct daughter sac arising from an already multilobulated aneurysm. Complex irregularity was assigned when a daughter sac was present on an already multilobulated aneurysm (Figure 1). The 50% threshold was used as a pragmatic operational imaging criterion to distinguish a focal protrusion from broader sac deformation and was not intended to represent a statistically derived prognostic cut-off.

Thus, the analysis included both an overall binary classification of aneurysm wall contour (regular versus irregular) and a feature-based assessment of specific irregularity patterns. Because daughter sac and multilobulated morphology could coexist, the percentages of individual morphological features were not expected to sum to the total percentage of irregular aneurysms.

This classification system was consistently applied across all cases using high-resolution radiological imaging (CTA or DSA) and standardized morphological criteria. Cumulative irregularity was analyzed as a distinct factor.

## 3. Results

### 3.1. Patient Characteristics

A total of 180 patients with 180 index aneurysms were included in the final analysis. The cohort consisted predominantly of older adults and women, with a substantial proportion of patients harboring multiple aneurysms. Subarachnoid hemorrhage was present in approximately one-fifth of patients, whereas the remaining cases represented unruptured aneurysms. The most frequent aneurysm locations were the middle cerebral artery, internal carotid artery, and anterior communicating artery. Overall, the cohort represented a clinically heterogeneous population of ruptured and unruptured intracranial aneurysms suitable for evaluating associations between aneurysm wall morphology, morphometric parameters, and established rupture- or growth-risk scores. Detailed demographic, clinical, anatomical, and risk-score characteristics are presented in Table 1.

### 3.2. Aneurysm Irregularity Is a Common Finding in Intracranial Aneurysms

Overall, irregular morphology was observed in 78 index aneurysms (43.3%). In the feature-based assessment, daughter sacs were present in 45 aneurysms (25.0%), multilobulated morphology in 65 aneurysms (36.1%), and complex irregularity in 20 aneurysms (11.0%). Because these morphological descriptors were not treated as mutually exclusive, the percentages of individual features do not sum to the total percentage of irregular aneurysms.

Paired interobserver ratings were available for 179 index aneurysms. Overall agreement for the four-category morphological assessment was 84.9% (152/179), with a Cohen’s kappa of 0.776, indicating substantial agreement. For the binary distinction between regular and irregular morphology, agreement was 92.2% (165/179), with a Cohen’s kappa of 0.844. The bootstrap 95% confidence interval for the four-category Cohen’s kappa was 0.695–0.848.

Mean size ratio was 2.77 ± 1.58; aspect ratio, 1.16 ± 0.56. The average perpendicular height was 4.41 ± 2.83 mm, with a mean volume estimated at 91.91 ± 217.00 mm^3^.

### 3.3. Irregular Wall Is Correlated with Higher Scores in PHASES and ELAPSS

**Figure 2:**
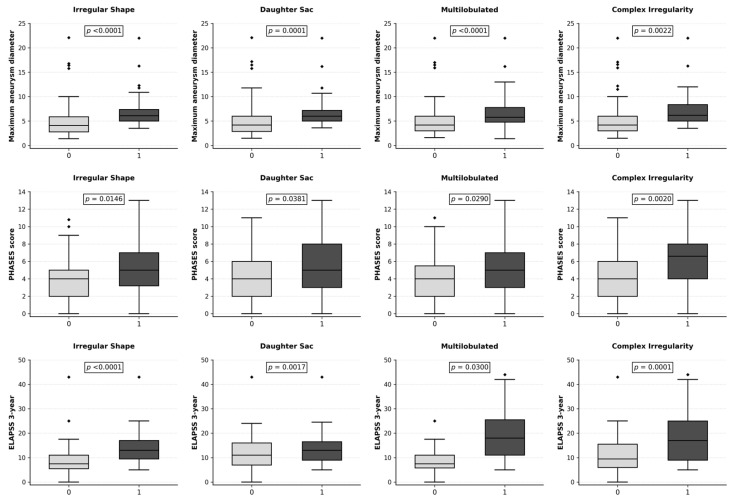
Box plots presenting differences in maximum aneurysm diameter, PHASES score, and ELAPSS 3-year growth risk according to the presence or absence of the analyzed morphological features. Grey boxes represent aneurysms without the analyzed feature (coded as 0), whereas black boxes represent aneurysms with the analyzed feature (coded as 1). The horizontal line within each box represents the median, boxes indicate the interquartile range, and whiskers represent the non-outlier range. Diamond-shaped points indicate outliers. The *p*-value was calculated using the Mann–Whitney U test.

The presence of a daughter sac was correlated with significantly higher PHASES scores (*p* = 0.0381) and higher 3-year (*p* = 0.0017) and 5-year (*p* = 0.0036) ELAPSS growth risks. No significant differences were observed for age (*p* = 0.3981), sex (*p* = 0.4421), or UIATS recommendation (*p* = 0.3314). Irregular shape was associated with higher PHASES (*p* = 0.0146) and ELAPSS (*p* < 0.0001) scores, as well as with UIATS treatment recommendations (*p* = 0.0006), but not with age or sex. Multilobulated morphology and complex irregularity also showed significant associations with PHASES and ELAPSS scores, but not with demographic variables or UIATS (Figure 2).

### 3.4. Propensity Score Matching Analysis Revealed Differences in the Frequency of Irregular Walls Between Ruptured and Unruptured Aneurysms

**Figure 3:**
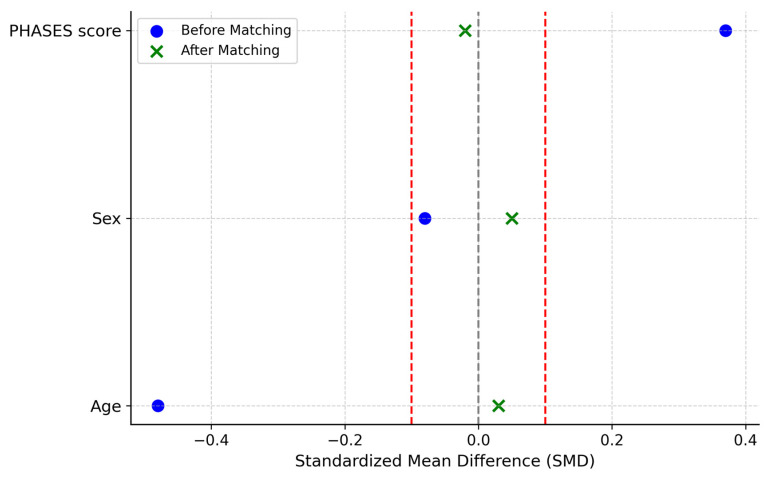
Standardized mean differences (SMDs) for covariates before and after propensity score matching (PSM), presented as a Love plot. Blue circles represent covariate balance before matching, whereas green crosses represent covariate balance after matching. The grey dashed vertical line indicates perfect balance (SMD = 0), and the red dashed vertical lines indicate the commonly used balance threshold of ±0.1. Lower absolute SMD values indicate better covariate balance between groups.

Initial analysis revealed that patients with SAH were significantly younger (*p* = 0.0006) and had higher PHASES scores (*p* = 0.0184). ELAPSS 5-year scores were numerically higher in the SAH group but did not reach statistical significance (*p* = 0.0954) (Table 2). Overall, irregular aneurysm shape was significantly more frequent in the SAH group than in the no-SAH group (62.5% vs. 37.9%, *p* = 0.0095). Individual morphological descriptors, including daughter sac, multilobulated morphology, and complex irregularity, were also numerically more frequent in the SAH group, but these differences did not reach statistical significance. To adjust for confounding, propensity scores were generated using age, sex, and PHASES and matched 1:1 using the nearest-neighbor method (caliper = 0.2 SD). After matching, 40 pairs were retained (*n* = 80), with no significant differences in baseline characteristics (Figure 3).

Following PSM, an irregular aneurysm shape was significantly associated with SAH. Irregular morphology was observed in 20/40 patients in the SAH group compared with 10/40 matched patients in the no-SAH group (50.0% vs. 25.0%, *p* = 0.0377), indicating an association between irregular morphology and rupture status in the matched cohort (Table 3).

### 3.5. Size-Adjusted Logistic Regression and Linear Regression Analyses

**Figure 4:**
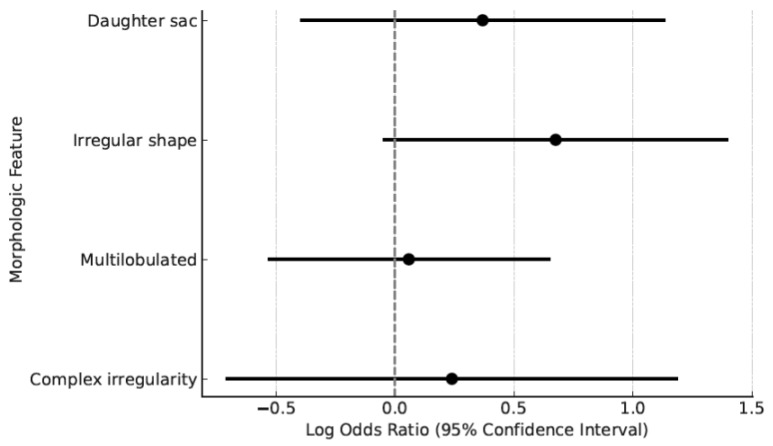
Size-adjusted logistic regression analysis of the association between aneurysm morphological features and subarachnoid hemorrhage. Points represent adjusted log odds ratios, and horizontal lines represent 95% confidence intervals. The vertical dashed line indicates the null effect line, corresponding to a log-odds ratio of 0 (odds ratio = 1). Confidence intervals crossing this line indicate no statistically significant association.

Logistic regression models were constructed to evaluate the association between aneurysm wall morphology and SAH while adjusting for maximum aneurysm diameter. The results are presented in Table 4 and Figure 4. Overall, the irregular shape showed a non-significant trend toward association with SAH (adjusted OR = 1.96; 95% CI: 0.96–4.04; *p* = 0.066). Individual irregularity descriptors were not significantly associated with SAH after adjustment for aneurysm size: daughter sac (adjusted OR = 1.45; 95% CI: 0.68–3.09; *p* = 0.340), multilobulated morphology (adjusted OR = 1.03; 95% CI: 0.50–2.13; *p* = 0.944), and complex irregularity (adjusted OR = 1.26; 95% CI: 0.44–3.61; *p* = 0.660). These findings suggest that the association between wall irregularity and rupture status may be partly explained by aneurysm size and should be interpreted as exploratory.

In the multivariable linear regression model, the presence of a daughter sac was associated with a 1.47 mm increase in maximum aneurysm diameter (95% CI: 0.33–2.62, *p* = 0.0118). Multilobulated configuration corresponded to the rise of 2.16 mm (95% CI: 1.16–3.17, *p* < 0.001), and complex irregularity to 2.40 mm (95% CI: 0.84–3.96, *p* = 0.0028). Irregular shape overall was associated with a 2.15 mm greater size (95% CI: 1.18–3.12, *p* < 0.001) (Table 5).

### 3.6. Multiple Factor Analysis and Clustering

**Figure 5:**
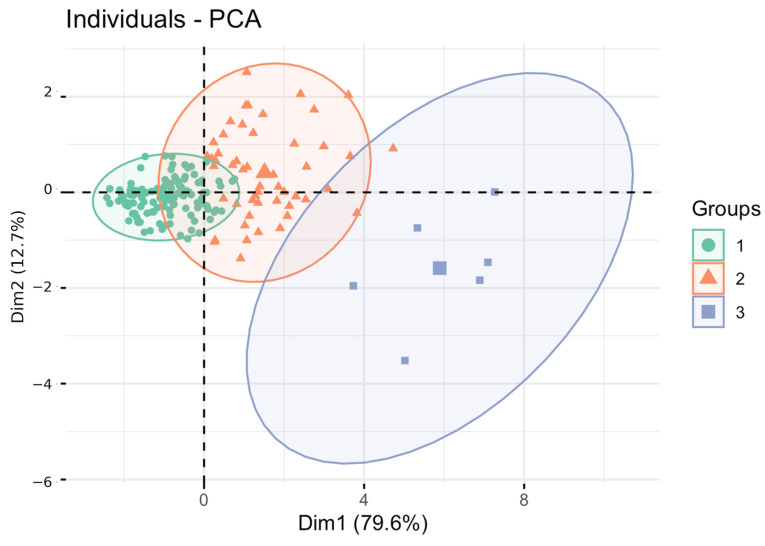
The first two principal components separate individuals into three clusters with differing bleeding risks, as assessed by the PHASES score. The model explains 92.3% of the variance. PCA—Principal Component Analysis. Cluster 1 included 120 cases, Cluster 2 comprised 52 cases, and Cluster 3 contained 6 cases. In Cluster 1, the most common morphological pattern was a regular shape (65%), followed by a daughter sac (21%). In Cluster 2, the predominant pattern was multilobulated morphology (44%), while in Cluster 3, complex irregularity was most frequent (50%).

GAM-based feature selection identified maximum diameter (*p* < 0.001), perpendicular height (*p* = 0.0005), neck diameter (*p* < 0.001), size ratio (*p* = 0.007), daughter sac (*p* = 0.03), and complex irregularity (*p* = 0.004) as most predictive of PHASES risk. PCA and hierarchical clustering identified three patient groups with differing median 5-year rupture risks: Cluster 1 (0.9%, *n* = 120), Cluster 2 (0.7%, *n* = 52), and Cluster 3 (4.2%, *n* = 6) (Figure 5). Fisher’s exact test across the three patient clusters showed significant differences in distribution for multilobulated morphology (*p* = 0.010) and overall irregular shape (*p* = 0.0036), whereas daughter sac (*p* = 0.12) and complex irregularity (*p* = 0.34) did not differ significantly between clusters. Two patients were excluded from PCA because of missing values in variables required for this analysis.

## 4. Discussion

In this retrospective single-center cross-sectional study, aneurysm wall irregularity was a frequent finding, present in nearly half of the analyzed intracranial aneurysms. Specific irregularity patterns, including daughter sac, multilobulated morphology, and complex irregularity, were associated with larger aneurysm dimensions and higher established rupture- or growth-risk estimates, particularly PHASES and ELAPSS scores. Overall irregular morphology was more common among ruptured aneurysms in the matched analysis, suggesting a relationship between wall irregularity and rupture status. However, after adjustment for aneurysm size, individual irregularity subtypes were not independently associated with SAH, indicating that their clinical relevance may be closely linked to aneurysm size and overall morphometric risk. These findings support the systematic description of aneurysm wall morphology while emphasizing the need for prospective validation before specific irregularity patterns can be considered independent predictors of rupture.

Previous studies have investigated the relationship between morphological irregularity and the risk of aneurysm rupture [11,12,13]. However, most have employed simplistic, binary descriptors such as the presence or absence of a daughter sac or general irregularity without further differentiation. Moreover, no standardized nomenclature exists, making comparisons between studies challenging [8,9,14].

Lindgren et al. used a dichotomous system, categorizing aneurysms as either “smooth” or “irregular,” thereby grouping minor protrusions with complex multilobulated forms [9]. Similarly, in the UCAS Japan study, a daughter sac was defined as any wall protrusion, though the classification of more complex morphologies was not clearly delineated. In that study, 18.9% of aneurysms had a daughter sac; this rose to 24.8% among treated cases. Applying the same criteria to our cohort yielded a comparable prevalence [8]. Sturiale et al. evaluated a cohort of 245 SAH patients and classified any irregular morphology under a single, undifferentiated category. They reported irregularity in 55.9% of cases, slightly higher than in our cohort, likely due to the exclusive inclusion of ruptured aneurysms [15].

This lack of consensus regarding the definition and classification of aneurysm irregularity represents a persistent limitation in the literature. It likely contributes to the observed variability in the reported prognostic significance of morphological features. Our analysis revealed recurring morphological patterns that are not adequately captured by current binary systems. Therefore, we propose a structured morphological assessment framework based on the overall aneurysm contour and feature-based irregularity descriptors. This approach may help standardize the description of aneurysm wall morphology and facilitate future studies evaluating its potential role in assessing rupture risk.

In our cohort, feature-based irregularity descriptors, including daughter sac, multilobulated morphology, and complex irregularity, were associated with higher PHASES and ELAPSS estimates. Because PHASES and ELAPSS are composite risk scores that include aneurysm size and location, these associations should be interpreted as alignment with established risk markers rather than as direct evidence of independent prognostic value. Overall, an irregular shape, but not the daughter sac alone, was associated with UIATS treatment recommendations. Moreover, aneurysms with irregular morphology were consistently larger across all dimensions, resulting in greater overall volume. In addition, each irregular feature was independently associated with increased aneurysm size, following a gradation from simple (daughter sac) to more complex forms (multilobulated, complex irregularity). However, none of the initial multivariable models demonstrated a statistically significant association between individual morphological features—such as daughter sacs, multilobulation, or complex irregularity—and the presence of SAH, although general irregularity approached significance. This suggests that the study may have been underpowered to detect such effects in the whole cohort. After propensity score matching, overall irregular morphology was more frequent among patients with SAH than among matched patients without SAH. However, multivariable logistic regression did not confirm a statistically significant independent association after adjustment for aneurysm size. Therefore, this finding should be regarded as an association observed in an exploratory matched analysis rather than proof that wall irregularity is an independent rupture predictor.

From a biological standpoint, aneurysm wall irregularities likely represent outward manifestations of underlying pathological processes, including chronic inflammation, extracellular matrix remodeling, and hemodynamic stress. High-resolution vessel wall imaging studies have consistently demonstrated that aneurysms with irregular morphology are more likely to exhibit wall enhancement—an imaging marker of inflammation, neovascularization, and mural instability [16]. Histological analyses support these findings, revealing that irregular aneurysms often exhibit dense inflammatory infiltrates, vasa vasorum proliferation, and degradation of structural proteins such as elastin and collagen [17,18]. Hemodynamically, regions with low wall shear stress are prone to endothelial dysfunction, infiltration of inflammatory cells, and activation of matrix metalloproteinases, which weaken the wall and promote asymmetric growth [19]. These pathobiological changes may result in morphological changes such as lobulations or blebs, which are captured in our classification of irregularity subtypes. Furthermore, intramural thrombus and microhemorrhages may create localized outward remodeling, further exacerbating shape irregularity and aneurysm enlargement [20]. Taken together, these mechanisms provide a plausible biological explanation for our finding that irregular wall morphology is associated with larger aneurysm size and elevated PHASES/ELAPSS scores, supporting its inclusion in individualized rupture-risk assessment.

From a clinical perspective, our findings suggest that aneurysm wall irregularity should not be described only as a binary feature but may benefit from a more structured morphological assessment. Distinguishing between daughter sac, multilobulated morphology, and complex irregularity may improve the consistency of radiological reporting and facilitate communication within multidisciplinary neurovascular teams. In patients with unruptured aneurysms, the presence of irregular morphology, particularly when accompanied by larger aneurysm size or higher PHASES/ELAPSS estimates, may support closer surveillance, more detailed individualized risk assessment, or consideration of additional imaging evaluation. However, because individual irregularity subtypes were not independently associated with SAH after adjustment for aneurysm size, these descriptors should be interpreted as complementary morphological markers rather than stand-alone indications for treatment. Their potential role in clinical decision-making should be validated in larger prospective multicenter cohorts before incorporation into existing risk stratification tools.

This study has several limitations. First, the relatively small sample size and the limited number of SAH events reduced the statistical power of multivariable logistic regression, particularly for individual irregularity subtypes. This may explain why overall irregularity was associated with SAH after propensity score matching, whereas individual morphological descriptors did not reach statistical significance after adjustment for aneurysm size. Second, this was a retrospective single-center study, and the cohort consisted predominantly of patients selected for treatment or presenting with SAH, which may limit generalizability and introduce selection bias. Exclusion of patients with incomplete imaging or missing variables required for risk-score calculation may have further contributed to this selection bias. Third, ruptured aneurysms may have undergone post-rupture morphological changes, limiting causal interpretation of wall irregularity as a predictor of rupture [21,22,23].

Additional limitations relate to missing data, retrospective UIATS calculation, and image assessment. Selected clinical variables, particularly smoking status and hypertension, were incompletely documented; missing values were not imputed, and analyses were performed using available-case or complete-case approaches depending on the variables required for each analysis. UIATS was calculated retrospectively; therefore, longitudinal components such as documented aneurysm growth and de novo aneurysm formation were scored only when clearly documented in prior imaging or medical records, potentially leading to conservative UIATS estimates. Hereditary conditions relevant to UIATS scoring, including autosomal dominant polycystic kidney disease, were identified from existing records, without systematic renal imaging, genetic testing, or dedicated screening. Although imaging assessments were performed independently and without access to calculated risk scores or treatment recommendations, complete blinding to rupture status could not be guaranteed because the imaging context of acute SAH may have been apparent in some cases. Finally, the 50% threshold used to distinguish daughter sac from multilobulated morphology was a pragmatic operational criterion and requires validation in future studies.

Despite these limitations, our study offers several strengths. We evaluated aneurysm wall irregularity in the context of established risk prediction tools used in real-world clinical decision-making. Moreover, we applied robust statistical methods to control for confounding and proposed a reproducible morphological classification system based on objective imaging features. The need for further validation is consistent with contemporary guideline-based and meta-analytic literature emphasizing individualized management of unruptured intracranial aneurysms [24,25]. It is also supported by earlier morphometric studies showing that aneurysm shape descriptors, including aspect ratio, quantified nonsphericity, and other morphology parameters, may contribute to rupture-risk assessment [26,27,28]. From a mechanistic perspective, the biological plausibility of aneurysm wall irregularity is further supported by reviews of aneurysm formation, growth, inflammation, and rupture biology [29]. Future validation should also consider longitudinal growth, as aneurysm enlargement remains an important clinically relevant outcome in unruptured aneurysm follow-up [30].

## 5. Conclusions

Collectively, our findings emphasize the clinical importance of detailed aneurysm wall morphology. The study revealed a common prevalence of aneurysmal irregularities. The developed classification correlated positively with the aneurysm size, PHASES, and ELAPSS scores. Refined morphological descriptors, such as daughter sac, multilobulated morphology, and complex irregularity, may provide additional information for individualized aneurysm assessment. However, their incremental prognostic value beyond established clinical and morphometric risk factors requires validation in larger, multicenter prospective cohorts before integration into existing risk models.

## Figures and Tables

**Table 1:** Demographics of the cohort. Morphological features were recorded as non-mutually exclusive descriptors; therefore, their percentages do not sum to the total number of irregular aneurysms. MCA—middle cerebral artery, ICA—internal carotid artery, ACoA—anterior communicating artery, BA—basilar artery, ACA—anterior cerebral artery.

Variable	Category	Value
Age	Mean ± SD	67.2 ± 12.1 years
Sex	Female	130 (72.2%)
	Male	50 (27.8%)
Multiple aneurysms	No	97 (53.9%)
	Yes	83 (46.1%)
Smoking status	No	44 (24.4%)
	Yes	23 (12.8%)
	Not determined	113 (62.8%)
Hypertension	No	44 (31.7%)
	Yes	95 (68.3%)
	Missing data	41 (22.8%)
Alcohol abuse	No	173 (96.1%)
	Yes	7 (3.9%)
Aneurysm location	MCA	73 (40.5%)
	ICA	50 (27.8%)
	ACoA	32 (17.8%)
	BA	11 (6.1%)
	ACA	10 (5.6%)
	Other	4 (2.2%)
Largest aneurysm dimension	Mean ± SD	6.02 ± 3.41 mm
Subarachnoid hemorrhage	Present	40 (22.2%)
5-year rupture risk, PHASES score	Mean ± SD	1.62 ± 2.44%
3-year aneurysm growth risk, ELAPSS	Mean ± SD	13.66 ± 9.32%
5-year aneurysm growth risk, ELAPSS	Mean ± SD	21.51 ± 13.24%
UIATS recommendation	Conservative management	49.5%
	Inconclusive	34.4%
	Aneurysm repair	16.1%
Aneurysm contour	Regular shape	102 (56.7%)
	Irregular shape	78 (43.3%)
Morphological feature	Daughter sac	45 (25.0%)
	Multilobulated morphology	65 (36.1%)
	Complex irregularity	20 (11.1%)

**Table 2:** Crude analysis of selected clinical and morphological features between the SAH and no SAH groups. SAH—subarachnoid hemorrhage.

Variable	SAH (Mean ± SD)	No SAH (Mean ± SD)	SAH (n/%)	No SAH (n/%)	Test	*p*-Value
Age	62.85 ± 11.11	68.92 ± 12.05	-	-	Mann–Whitney U	0.0006
Female sex	-	-	32/40 (80.0%)	98/140 (70.0%)	Chi^2^	0.2959
Active smoking	-	-	3/11 (27.3%)	20/56 (35.7%)	Fisher’s exact test	0.7359
Hypertension	-	-	28/36 (77.8%)	67/103 (65.0%)	Chi^2^	0.2281
Alcohol abuse	-	-	3/40 (7.5%)	4/140 (2.9%)	Fisher’s exact test	0.1847
PHASES	5.24 ± 2.62	4.25 ± 2.80	-	-	Mann–Whitney U	0.0184
ELAPSS 5-year	23.02 ± 12.25	21.24 ± 13.46	-	-	Mann–Whitney U	0.0954
Irregular shape	-	-	25/40 (62.5%)	53/140 (37.9%)	Chi^2^	0.0095
Daughter sac	-	-	14/40 (35.0%)	31/140 (22.1%)	Chi^2^	0.1473
Multilobulated	-	-	17/40 (42.5%)	48/140 (34.3%)	Chi^2^	0.4429
Complex irregularity	-	-	6/40 (15.0%)	14/140 (10.0%)	Chi^2^	0.5471

**Table 3:** Analysis of the selected clinical and morphological features after Propensity Control Matching, controlling for age, sex, and PHASES score. SAH—subarachnoid hemorrhage.

Variable	SAH (Mean ± SD)	No SAH (Mean ± SD)	No SAH (n/%)	SAH (n/%)	Test	*p*-Value
Age	63.35 ± 10.71	63.87 ± 13.08	-	-	Mann–Whitney U	0.6113
Sex	-	-	30/40 (75.0%)	27/40 (67.5%)	Chi^2^	0.6231
Active smoking	-	-	14/32 (43.8%)	5/17 (29.4%)	Chi^2^	0.5012
PHASES	4.95 ± 2.35	4.97 ± 2.27	-	-	Mann–Whitney U	0.9596
ELAPSS 5-year	21.29 ± 11.31	20.54 ± 12.86	-	-	Mann–Whitney U	0.3186
Irregular shape	-	-	10/40 (25.0%)	20/40 (50.0%)	Chi^2^	0.0377
Daughter sac	-	-	6/40 (15.0%)	11/40 (27.5%)	Chi^2^	0.2743
Multilobulated	-	-	11/40 (27.5%)	14/40 (35.0%)	Chi^2^	0.8113
Complex irregularity	-	-	3/40 (7.5%)	5/40 (12.5%)	Chi^2^	0.7094

**Table 4:** Size-adjusted logistic regression analysis evaluating the association between aneurysm wall morphology and subarachnoid hemorrhage. Separate logistic regression models were fitted for each morphological feature and adjusted for maximum aneurysm diameter. OR—odds ratio; CI—confidence interval; SAH—subarachnoid hemorrhage.

Morphological Feature	Adjusted OR	95% CI	*p*-Value
Daughter sac	1.45	0.68–3.09	0.340
Irregular shape	1.96	0.96–4.04	0.066
Multilobulated morphology	1.03	0.50–2.13	0.944
Complex irregularity	1.26	0.44–3.61	0.660

**Table 5:** Linear regression analysis evaluating the association between individual morphological features and maximum aneurysm diameter. Coefficients represent the mean difference in aneurysm size (in millimeters) associated with the presence of each feature. Confidence intervals (CI) are reported at the 95% level.

Feature	Coefficient (mm)	95% CI	*p*-Value
Daughter sac	1.47	0.33–2.62	0.0118
Multilobulated morphology	2.16	1.16–3.17	<0.001
Complex irregularity	2.40	0.84–3.96	0.0028
Irregular shape	2.15	1.18–3.12	<0.001

## Data Availability

The original contributions presented in this study are included in the article. The datasets generated and/or analyzed during the current study are available from the corresponding author on request, due to institutional policies concerning the sharing of retrospective clinical data. Further inquiries can also be directed to the corresponding author.

## Abbreviations

The following abbreviations are used in this manuscript:

ACA	anterior cerebral artery
ACoA	anterior communicating artery
BA	basilar artery
CI	confidence interval
CTA	computed tomography angiography
DSA	digital subtraction angiography
ELAPSS	Estimated aneurysm Lifetime, Age, Population, Size and Site score
GAM	generalized additive model
ICA	internal carotid artery
MCA	middle cerebral artery
PCA	principal component analysis
OR	odds ratio
PHASES	Population, Hypertension, Age, Size of aneurysm, Earlier SAH, Site score
PSM	propensity score matching
SAH	subarachnoid hemorrhage
SD	standard deviation
SMD	standardized mean difference
UIATS	Unruptured Intracranial Aneurysm Treatment Score
